# Expression of toll-like receptors 2 and 4 in subjects with asthma by total serum IgE level

**DOI:** 10.1186/s12931-016-0355-2

**Published:** 2016-04-16

**Authors:** Astrid Crespo-Lessmann, Eder Mateus, Silvia Vidal, David Ramos-Barbón, Montserrat Torrejón, Jordi Giner, Lorena Soto, Cándido Juárez, Vicente Plaza

**Affiliations:** Respiratory Department, Hospital de la Santa Creu i Sant Pau & Biomedical Research Institute Sant Pau (IIB Sant Pau), Sant Antoni Maria Claret 167, 08025 Barcelona, Spain; Department of Medicine, Universitat Autònoma de Barcelona, Barcelona, Spain; Immunology Department, Hospital de la Santa Creu i Sant Pau & Biomedical Research Institute Sant Pau (IIB Sant Pau), Barcelona, Spain

**Keywords:** Asthma, Toll-like receptors, Innate immunity, IgE

## Abstract

**Background:**

Emerging data suggest that innate immunity may play a role in asthma, particularly the toll-like receptors (TLRs). Some studies pointed to an involvement of TLRs 2 and 4 in the pathogenesis of allergic asthma, and other studies related TLRs to IgE. However, there are not any studies that have comprehensively evaluated the expression of TLRs 2 and 4 in inflammatory cells, in peripheral blood and induced sputum specimens from asthmatic patients, according to their total serum IgE.

**Methods:**

We studied 44 asthmatic patients (15 with high total serum IgE and 29 with normal total serum IgE). On a single visit, all patients underwent: induced sputum, pulmonary function tests, determination of exhaled nitric oxide fraction, venipuncture for blood analysis and skin prick allergy tests. The induced sputum cellularity was analyzed by flow cytometry, where expression of TLRs 2 and 4 was studied using fluorochrome-conjugated monoclonal antibodies.

**Results:**

Asthmatic patients with high total serum IgE showed, a higher percentage of macrophages expressing TLR4 (42.99 % ± 22.49) versus asthmatic patients with normal total serum IgE (28.84 % ± 15.16) (*P* = 0.048). Furthermore, we observed a correlation (but weak) between the percentage of macrophages expressing TLR4 in induced sputum and the total serum IgE level (*R* = 0.314; *P* = 0.040).

**Conclusion:**

Asthmatic subjects with high total serum IgE show increased macrophage expression of TLR4 in induced sputum. This outcome may result from a link between innate immunity and IgE-mediated, adaptive immune responses in asthma, and point to TLR4 as a potential therapeutic target.

## Background

IgE plays a prominent role in the pathophysiology of allergic asthma. IgE binds to receptors on the surface of different types of immune effector cells causing them to release a variety of mediators that promote airway hyperresponsiveness, mucus secretion and increased vascular permeability [1]. Several strategies for decreasing IgE have been developed as a possible treatment for asthma [1]. Some studies have reported that the ligation of Toll-like receptors (TLRs) by bacterial or viral antigens can affect IgE-dependent mast cell degranulation and release of preformed mediators, as well as eicosanoid production, thus providing evidence on the involvement of innate immunity in the pathogenesis of asthma [2–5]. Among the various agents involved in innate immunity in the pathogenesis of asthma, the TLRs may bear an important role [6–10].

TLRs are a family of cell surface proteins involved in the recognition of pathogen-associated molecular patterns (PAMPs). Upon stimulation, TLRs can modulate subsequent adaptive immune responses. TLR2 and 4 are expressed on the cellular surface and migrate to phagosomes after activation on recognising the ligand. Just because these receptors are expressed on the cellular surface makes them easy to measure. Our laboratory has expertise in measuring them [6].

TLRs 2 and 4 function as signal receivers for gram-positive and negative bacteria (endotoxin recognition) [6–8]. In some studies, a relationship between microbial products and regulation of the T helper (Th)1 and Th2 responses through TLRs 2 and 4 was observed in allergic diseases [11–13]. Interestingly, TLRs 2 and 4 can modulate the Th response according to the bacterial burden, where a high lipopolysaccharide (LPS) level induces a Th1 response and a low LPS level promotes a Th2 response [9, 14, 15]. Furthemore, there is evidence that exposure to endotoxin during early life [16, 17] may be protective against the development of atopy and asthma, an argument in support of the hygiene hypothesis, although such association remains poorly understood [17].

Previous studies addressing the role of TLRs 2 and 4 in asthmatic patients had several limitations. No studies approached a differential analysis of TLRs 2 and 4 in blood versus induced sputum. Most of the studies were limited to blood cells (frequently monocytes) [18, 19], in experimental asthma models or in cell cultures [9, 13]. The few studies conducted in humans were done on diseases other than asthma, such as COPD, bronchiectasis, and cystic fibrosis [19–23]. The induced sputum technique is a noninvasive procedure that provides direct information on cells and mediators involved in airway inflammation. Thus, we expected induced sputum analysis to be particularly suited to study the role of the innate immune response in the pathogenesis of asthma. The main objective of the present study was to assess the expression of TLRs 2 and 4 in monocytes/macrophages and neutrophils, both from peripheral blood and induced sputum, in a group of adult asthmatic patients stratified by their level of total serum IgE.

## Methods

The study was approved by the Institutional Review Board and registered at ClinicalTrials.gov with identifier NCT02028637.

### Legal and ethical aspects

The study was conducted in accordance with the Declaration of Helsinki principles (18th Word Medical Assembly, 1964) and was approved by the Clinical Research Ethics Committee (approval number: IIBSP/43/2009) of our institution. The participants signed their informed consent to participate in this study and personal identification data were anonymized.

### Subjects and study design

This was an observational, cross-sectional study performed at a hospital outpatients clinic. Forty-four subjects on maintenance treatment for asthma, aged 18 to 75 years, were included. All subjects were non-smokers on study inclusion and had asthma diagnosed as per the Global INitiative for Asthma (GINA) criteria [24]. Furthermore, these patients received inhaled corticosteroids according to GINA [24]. Asthmatic patients were defined as having high total serum IgE when they had a ≥160 IU/mL total IgE value in a peripheral blood sample [25]. A normal level of total serum IgE was defined as less than 160 IU/mL [25]. Patients were excluded if they had: (i) a respiratory tract infection and/or required the use of oral corticosteroids within 30 days prior to inclusion; (ii) immunomodulatory treatment (iii) low adherence to treatment; (iv) any pulmonary pathology other than asthma or a significant comorbidity that might affect the study results upon physician’s judgment; or (v) a cognitive impairment that could limit their understanding or collaboration in the study.

### Clinical procedures

All study procedures were performed at a single clinic visit. After signing the informed consent, the participating patients fulfilled an Asthma Control Test (ACT) questionnaire [26] and underwent spirometry, induced sputum collection, measurement of exhaled nitric oxide fraction (FeNO), peripheral venous blood sampling, and skin prick test with standardized allergen extracts (based on the modified test of Pepys) [27]. Spirometry was performed with a Datospir 500 (Sibelmed S.A., Barcelona, Spain) following the standards of the European Respiratory Society (ERS) [28] and the *Sociedad Española de Neumología y Cirugía Torácica* (SEPAR) [29]. FeNO was measured with a chemiluminescence (SIR® N-6008 device, Madrid, Spain) according to established standards [30] and reference values [31]. Total serum IgE was measured by enzyme-linked immunoassay (ImmunoCAP, Phadia 250. Phadia AB, Uppsala, Sweden). Normal values of total IgE were established by the laboratory [25]. Induced sputum samples were harvested according to the ERS consensus protocol [32] and processed for flow cytometry and conventional readouts. Briefly, sputum induction was performed using an inhalation of an aerosol of hypertonic saline at increasing concentrations (3, 4 and 5 %) generated by an ultrasonic nebulizer (Omron NE U07, HEALTHCARE Europe, Germany) with an output of 3 ml/s and particle size of 7 μm aerodynamic mass median diameter. Sputum processing was initiated from the fresh specimens within two hours.

### Processing of sputum and blood specimens

Induced sputum specimens were processed according to a consensus standard procedure [33]. Mucus plugs were manually selected and weighed, incubated for 15 min at room temperature in 0.1 % dithiothreitol (DTT) (Calbiochem, San Diego, CA) in phosphate-buffered saline (PBS) into a total mL volume of four-fold the weight in mg of the selected plug, and then washed and gravity filtered through a 41-μm pore nylon mesh (Millipore, Membrane solutions, Dallas, Tx, USA). After homogenization with DTT, each specimen was aliquoted into two portions of equal volume, one to be processed for conventional microscopic examination and the other for flow cytometry analysis. Total cell counts were done in a Neubauer hemacytometer, and cytocentrifuged slides stained with Diff-Quik kit (Polysciences Europe GmbH, Eppelheim, Germany) were used for differential leukocyte counts. Squamous epithelial cells were excluded from the total cell count and were required to be less than 20 % of the total cells as specimen quality criterion. Samples containing less than 10^6^ cells/g were not included in the analysis. Cell viability was determined by trypan blue dye exclusion and was required to be greater than 40 % as quality criterion. Differential leukocyte counts were done on a minimum of 400 cells and were expressed as cell percentage of lymphocytes, neutrophils, eosinophils and macrophages. Cell count reference values were previously established [34]. The sputum cell suspension for flow cytometry analysis was delivered into 100-μl/tube samples approximately containing 10 [5] cells/tube.

Peripheral venous blood was treated with trisodium citrate as anticoagulant, then aliquoted into 100 μl/tube samples, and erythrolysed with TQ Prep Sample Preparation and COULTER PrepPlus 2 (Beckman Coulter, Miami, Florida), and immunostaining for flow cytometry followed.

### Flow cytometry

Sputum and blood cell suspensions were blocked with mouse serum and immunostained with saturating concentrations of the following fluorochrome-conjugated monoclonal antibodies, for 15 min at room temperature in the dark: phycoerythrin (PE) anti-TLR4 (clone HTA125), PE anti-CD66b (G10f5) and PE/Cyanin-7 anti-CD14 (clone M5E2) from Biolegend (San Diego, California); Alexa Fluor 488 anti-TLR2 (clone 11G7) and PE anti-CD125 (A14) from BD Biosciences (Eembodegen, Belgium); PE anti-CD16 (3G8), PE anti-CD45 (MEM28), fluorescein isothiocyanate (FITC) anti-CD16 (3G8), FITC anti-CD66b (B13.9), and PE/Dy-647 anti CD45 (MEM28) from Immunotools (Oldenburg, Germany) [33]. PE mouse IgG2a, ƙ clone MOPC-173 and FITC mouse IgG1, ƙ clone MOPC21 were used to test the specificities of TLR4 and TLR2 antibodies. The cells were then washed with 2 mL of staining buffer (1 % bovine serum albumin in PBS) and centrifuged for 5 min at 400 G. Supernatants were decanted, the cells resuspended in 300 μL of staining buffer, and the samples stored at 4 °C in the dark until analyzed through the flow cytometer within 2 h.

Flow cytometry data acquisition was performed with a FC500 equipment (Beckman Coulter, Pasadena, California, USA). Ten thousand events were analyzed for all sample runs. Gating of sputum leukocytes was based on side light scatter versus CD45 expression, which allowed for the discrimination of lymphocyte, macrophage and granulocyte populations. The mean fluorescence intensity (MFI) of the cells stained with control antibody was subtracted from the MFI of the cells stained with receptor antibodies to provide a measure of receptor-specific fluorescence.

### Classification of asthma inflammatory phenotypes

Asthma inflammatory phenotypes were classified according to sputum cellularity. Patients were classed as neutrophilic asthma if the neutrophil count was >61 %, eosinophilic asthma if eosinophils >3 %, as and those with <61 % neutrophils and <3 % eosinophils were considered paucigranulocytic asthma [35].

### Statistical analysis

Values are presented as percentages and frequencies for qualitative data and mean ± standard deviation for quantitative data. Comparisons between asthma with high versus normal total serum IgE were analyzed with Student’s t-test. Categorical variables were contrasted through contingency tables and tested with chi-square, or Fisher’s test where appropriate. Pearson’s coefficient was employed for correlation analysis. The level of statistical significance was set as α = 0.05. Analysis was done with SPSS software version 18.0 for Windows (SPSS, Inc., Chicago, Il, USA).

## Results

### Clinical outcomes and asthma phenotypes

Out of the 44 subjects with asthma studied, 15 had high total serum IgE and 29 had normal total serum IgE. Demographics, clinical and functional data are shown in Table 1. No significant differences were observed between both groups except for the levels of total serum IgE and skin prick test results. As for the latter, 69 % of the asthmatic patients with high level of total IgE were sensitized to house dust mites, 23 % to various pollens, and 8 % to fungi species. Among the subjects classed as asthmatic patients with a normal level of total IgE, 53.57 % showed a positive skin prick test (60 % to house dust mites, 13 % to pollens and 27 % to other allergens). Data from induced sputum quality assessment and cell counts are summarized in Table 2. Induced sputum quality was high in most samples as per the cell content, cell viability and percentage of squamous cells criteria, with no significant differences between groups. Sputum differential leukocyte counts revealed the paucigranulocytic asthma phenotype as the most common in both asthma groups, followed by the eosinophilic phenotype in the asthmatics with high total IgE and the neutrophilic phenotype in the asthmatics with normal IgE respectively, yet no significant differences were found between both asthma groups in terms of inflammatory phenotype classification.

**Table 1:** Demographics, clinical and pulmonary function data

	Normal total serum IgE (*n* = 29)	High total serum IgE (*n* = 15)	*P*
Age (years), mean (STD)	51.34 (17.5)	52.06 (15.6)	0.894
Sex (% women)	74 %	52.94 %	0.150
BMI (kg/m^2^), mean (STD)	26.59 (4.45)	27.87 (4.3)	0.368
Rhinitis, % subjects (n)	62.06 % (18)	80 % (12)	0.194
Nasal polyposis, % subjects (n)	21.7 % (6)	40 % (6)	0.157
ACT ≥ 20, % subjects, (n)	58.62 %	73.33 %	0.267
FEV1%, mean (STD)	84.93 (18.4)	77.13 (20.7)	0.209
FEV1/FVC, mean (STD)	68.8 (13.9)	63.29 (11.8)	0.173
Total IgE (UI/mL), mean (STD)	67.5 (44.7)	545.4 (467.8)	0.000
Positive skin prick test, % subjects)	55.17 %	86.66 %	0.036
FeNO (ppb), mean (STD)	39.3 (34.1)	28.8 (17.2)	0.272
Patients receiving 2 or > OC within last 12 m, % subjects	1.85 (2.8)	2.33 (3.9)	0.576
ICS dose in patients (high ICS dose: ≥ 800 μg/day Beclomethasone or equivalent), % subjects (n)	31.03 % (9) H 34.5 %(10) M 31.03 % (9) L 3.44 % (1) N	27 %(4) H 27 % (4) M 46 % (7) L 0 % (0) N	0.697

Values are mean ± standard deviation (STD) or percentage and number of subjects (n), as indicated

*BMI* body mass index, *ACT* Asthma Control Test, *FEV*_*1*_ forced expiratory volume in first second, *FVC* forced vital capacity, *IgE* total immunoglobulin E, *FeNO* exhaled nitric oxide fraction, *OC* oral corticosteroids, *ICS* inhaled corticosteroids, *H* high ICS dose, *M* medium ICS doses, *L* low ICS dose, *N* do not use CIS

**Table 2:** Inflammatory phenotypes, total and differential leukocyte counts in induced sputum and specimen quality parameters, distributed as per total serum IgE

	Normal total serum IgE (*n* = 29)	High total serum IgE (*n* = 15)	*P*
Eosinophilic phenotype, % (n)	17.24 (5)	20 (3)	0.620
Neutrophilic phenotype, % (n)	20,68 (6)	13.33 (2)	0.380
Paucigranulocytic phenotype, % (n)	62 (18)	66,66 (10)	0.421
Neutrophils in sputum, % (STD)	49.54 (22.31)	39.93 (20.7)	0.173
Eosinophils in sputum, % (STD)	5.68 (4.96)	10.61 (12.33)	0.065
Lymphocytes in sputum, % (STD)	0.83 (0.78)	0.76 (0.46)	0.769
Macrophages in sputum, % (STD)	40.71 (21.95)	48.38 (21.51)	0.278
Cellular concentration of the sputum sample (× 10^6^ cell/g), mean (STD)	2.56 (1.81)	3.53 (2.46)	0.349
Sample cell viability, % (STD)	58.91 (20.82)	49.00 (20.04)	0.406
Epithelial cells, % (STD)	5.61 (8.49)	5.53 (5.18)	0.781

Values are percentage and number of subjects (n), mean percentage and standard deviation (STD), or mean and STD as indicated

### TLR expression

TLR expression data are shown in Table 3. TLR2 was mostly expressed in monocytes/macrophages, and to a lesser extent in neutrophils, in both induced sputum and peripheral blood. No significant differences were observed between both groups in the percentage of cells expressing TLR2 in either induced sputum or blood, nor in the mean of fluorescence intensity in the cells analyzed.

**Table 3:** Expression of TLRs 2 and 4 distributed as per total serum IgE

	Normal total serum IgE (*n* = 29)	High total serum IgE (*n* = 15)	*P*
Percentage of cells expressing TLR2			
Neutrophils (sputum)			
● Cells (%)	0.89 (0.97)	2.81 (5.02)	0.180
● MFI	2.78 (2.24)	2.81 (1.26)	0.954
Macrophages (sputum)			
● Cells (%)	30.78 (21.91)	31.7 (26.36)	0.907
● MFI	2.18 (0.42)	2.55 (0.73)	0.103
Neutrophils (blood)			
● Cells (%)	1.19 (0.51)	1.36 (1.32)	0.637
● MFI	1.77 (0.68)	1.77 (0.64)	0.974
Monocytes (blood)			
● Cells (%)	85.54 (10.42)	82.61 (15.14)	0.510
● MFI	2.47 (0.59)	2.71 (0.98)	0.379
Percentage of cells expressing TLR4			
Neutrophils (sputum)			
● Cells (%)	2.96 (2.7)	7.64 (11.08)	0.118
● MFI	3.83 (2.91)	4.9 (5.34)	0.157
Macrophages (sputum)			
● Cells (%)	28.84 (15.16)	42.99 (22.49)	0.048
● MFI	13.18 (11.41)	13.13 (14.39)	0.990
Neutrophils (blood)			
● Cells (%)	2.13 (2.12)	1.74 (1.55)	0.532
● MFI	2.66 (1.41)	2.51 (0.72)	0.576
Monocytes (blood)			
● Cells (%)	61.81 (14.42)	65.41 (17.00)	0.468
● MFI	2.89 (0.93)	2.56 (0.97)	0.281

*MFI* Mean of Fluorescence Intensity, *TLR* toll-like receptors. Values are mean percentage and standard deviation (STD) for cell counts, and mean (STD) for MFI

**Fig. 1:**
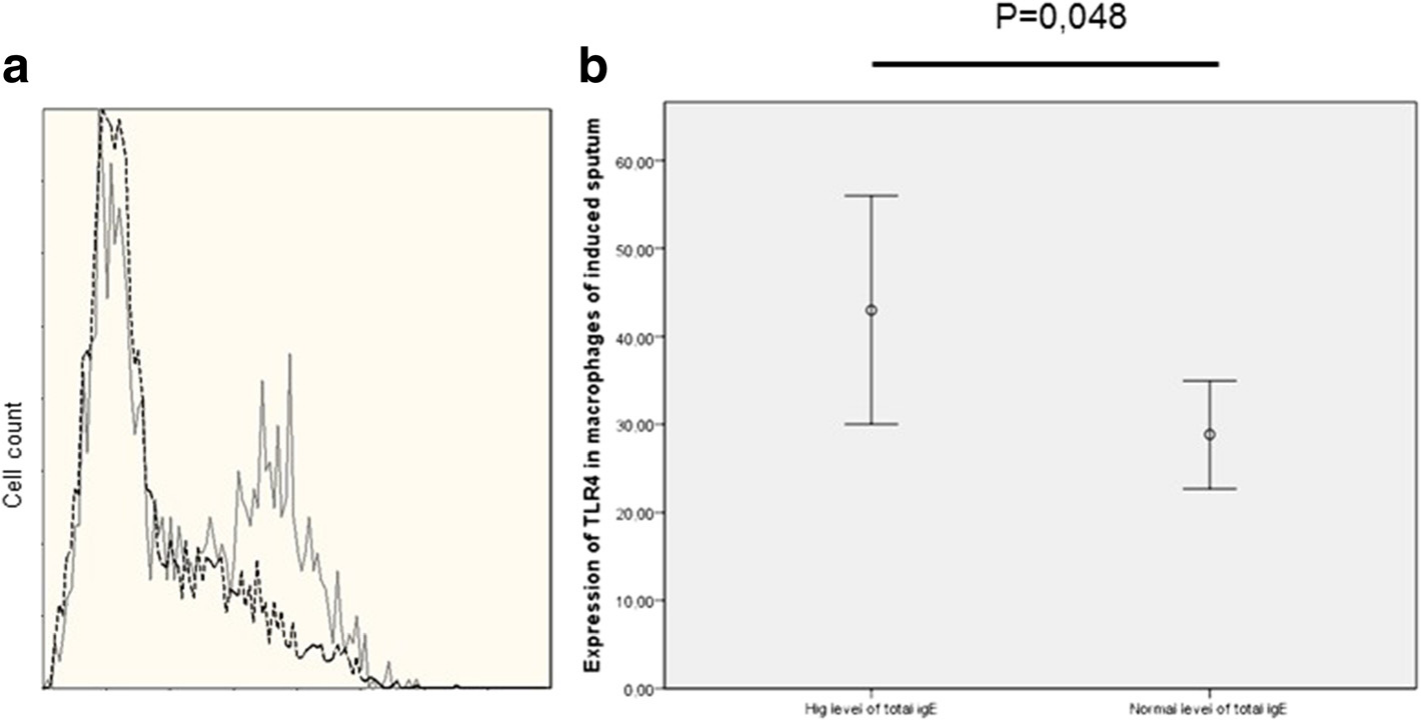
Expression of TLR4 in macrophages from induced sputum. Macrophages were gated within the CD45^+^ cells and identified as CD14^+^CD66b. **a** Example of TLR4 expression by induced sputum macrophages in asthmatic subjects with high total serum IgE (dotted histogram) versus asthmatic patients with normal IgE level (grey line histogram). **b** Expression of TLR4 in macrophages of induced sputum in asthmatic subjects with normal versus high level total serum IgE (values are mean percentage and 95 % confidence intervals)

The percentage of macrophages expressing TLR4 in induced sputum was significantly higher in asthma with high total serum IgE versus asthma with normal total serum IgE (Fig. 1). In both asthma groups, a greater percentage of TLR4 expression was observed in monocytes/macrophages than neutrophils in both induced sputum and peripheral blood. In the rest of leukocyte subpopulations, there was no significant difference in the percentage of cells expressing TLR4, nor in the mean of fluorescence Intensity.

**Fig. 2:**
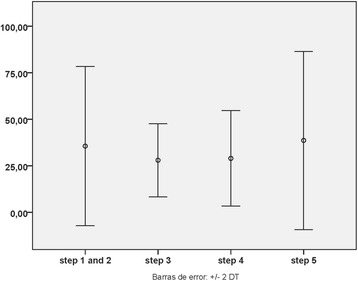
Expression of TLR4 by induced sputum macrophages as per GINA maintenance therapy step

The percentage of TLR4^+^ macrophages was not influenced by the patient’s GINA therapeutic step (Fig. 2; 35.59 % (21.39) [mean percentage (STD)] in steps GINA-1 and 2 together, *n* = 8; 28.01 % (9.82) in GINA-3, *n* = 11; 28.99 % (12.83 %) in GINA-4, *n* = 9; and 38.66 % (23.93) in GINA-5, *n* = 16; *P* = 0.501).

### Correlations

We observed a correlation (but weak) between total serum IgE and the percentage of macrophages expressing TLR4 in induced sputum (R=0.314, P=0.04). A correlation was also found between the percentage of macrophages and neutrophils expressing TLR4 in induced sputum (R=0.432, P=0.008).

## Discussion

The main outcome of the present study was that the percentage of macrophages expressing TLR4 in induced sputum was higher in asthmatic patients with a high total serum IgE than in asthmatics with normal total serum IgE. Furthermore, we observed a correlation (but weak) between total serum IgE and the percentage of macrophages expressing TLR4 in induced sputum. This result is consistent with a possible link between innate immunity and the adaptive immune response in the pathogenesis of IgE-mediated asthma. The role of TLRs in the inflammatory process has been the subject of recent investigations. However, most of the studies have evaluated cellular responses in vitro or systemic reactions, and few studies focused on the role of TLRs in lung inflammation. In the present study, we analyzed the cellular expression of TLRs 2 and 4 on concurrent induced sputum and peripheral blood samples in adult asthmatics, and the data were stratified according to the type of asthma in terms of high versus normal level of total serum IgE. The study was limited to an observational assessment of TLR expression, yet the work was performed on a large sample of clinically well characterized patients, where all data were collected together at a single time point.

TLRs are a family of proteins responsible for the recognition of PAMPs, which include repetitive microbial molecular domains such as lipopolysaccharides, flagellin, mannose and nucleic acids from viruses and bacteria. TLRs 2 and 4 are expressed on the cell surface and translocate to phagosomes after activation, upon binding their ligands. TLR4 activation leads to a series of events including bronchoconstriction, expression of adhesion molecules by vascular endothelial cells and the release of cytokines. Such actions result in neutrophil recruitment and activation of pulmonary dendritic cells and macrophages [36], as well as a Th2 polarization of subsequent adaptive immune responses [37]. Previous studies showed that, in genetically susceptible subjects, allergen exposure along with low PAMP doses favors allergic responses [38]. In contrast, high-dose PAMP exposure, as it occurs in livestock farms, rural environments in developing countries and traditional lifestyles, leads to antigen tolerance [39]. Such protective “farm effect” has been attributed to an immunomodulatory role of the exposure to LPS and other TLR ligands during early childhood, which results in inhibiting the development of allergic immune responses [9, 40–42]. Some recent studies [6] have led to interesting expectations by observing that in neutrophilic asthma there is an increase in the expression TLR2, TLR4, CD14 and surfactant protein-A (SP-A), and that the activation of TLR, by an allergen for example, generates a cascade of signals driven by the activation and nuclear translocation of NF-kB, that results in a cytokine-mediated inflammatory response. The discovery of TLR and their actions provides an immunological basis for the study of the hygiene hypothesis. In this study, we measure the TLR2 and 4 for several reasons: 1) because they are receptors expressed on the cell surface and they are easy to measure 2) because in our laboratory we have more experience in the measurement of receptors expressed on the cell surface and 3) because these receptors are associated in the pathogenesis of asthma, specifically with the hygiene hypothesis and the respiratory infections [16, 17]. In this study, none of the patients included had a respiratory infection within the previous month, nor suffered from bronchiectasis.

There are findings showing that bacterial and viral infections can modify the course of allergic diseases by affecting high-affinity IgE receptor (FcεRI)-dependent mast cell activation. Such studies reported that the ligation of TLRs by bacterial or viral antigens can affect IgE-dependent mast cell degranulation and preformed mediator release, as well as eicosanoid production [2]. Furthermore, it has been reported that a synergistic interaction between TLR ligands and allergens can also modify cytokine synthesis by mast cells stimulated via their high-affinity IgE receptor, FcεRI [3–5]. Our present findings showing higher numbers of induced sputum macrophages expressing TLR4 in asthmatic subjects with high total serum IgE, along with a correlation (weak) between the percentage of TLR4^+^ macrophages and the total serum IgE level, are consistent with the idea of a relationship between respiratory exposure to PAMPs and asthma with increased IgE, and suggest an involvement of the macrophage in such relationship.

Macrophages are key cells in the pulmonary innate immune responses, since they are the most abundant leucocyte in the air spaces and one of the first cells to encounter inhaled proteins that may act as allergens. Depending on the signals received, macrophages can be pro- or anti-inflammatory. Macrophage stimulation can result from a variety of stimuli including TLR engagement in the presence of IL-10 or other cytokines [37]. Our analysis of TLR 2 and 4 expression by different inflammatory cells types in induced sputum and blood showed a higher expression of these receptors in monocytes/macrophages than in neutrophils, both in sputum and peripheral blood, which may reflect the relevance of the macrophage in the pulmonary innate immune responses. Our findings are also consistent with previous studies showing that TLR4 stimulation can induce further recruitment of macrophages to the airways [37].

The role of TLR2 in airway allergic inflammation is not fully clear. In our work we did not find any significant relationship between TLR2 expression and total serum IgE. However, TLR2 stimulation by its ligands was reported to potentiate Th2 responses and exacerbate airway hyperresponsiveness [43, 44]. The relationship between TLR2 activation and allergic responses is complex and may depend on the antigen nature and dose, the timing, and the TLR associated ligands [45]. Variability in the techniques employed, and the cell populations and specimens analyzed, may also account for limitations in the comparability among different studies.

Our study has some limitations: (i) the effect of inhaled corticosteroids on expression of TLRs in induced sputum is unknown; (ii) we only analyze receptors that are expressed on the cell surface (TLR4 and 2); (iii) it is a descriptive study. Our results were significant but, should be studied with a higher population size.

## Conclusion

In summary, this work is the first evaluation of the expression of TLRs by blood and induced sputum leukocytes in subjects with asthma. The main finding was that asthmatic patients with a high total serum IgE have a higher percentage of macrophages expressing TLR4 in induced sputum, compared with patients with normal total serum IgE values. This outcome supports the possibility of therapeutic approaches for some forms of immune-mediated lung disease through stimulating or blocking TLRs with agonists or antagonists [46], and may be exploited in the future for therapeutic target discovery.
